# N1 and N2 neutrophil subtypes in breast cancer: functional implications and clinical perspectives: a narrative review

**DOI:** 10.1097/MS9.0000000000003609

**Published:** 2025-07-18

**Authors:** Emmanuel Ifeanyi Obeagu

**Affiliations:** Department of Biomedical and Laboratory Science, Africa University, Mutare, Zimbabwe

**Keywords:** breast cancer, immunotherapy, N1 neutrophils, N2 neutrophils, tumor microenvironment

## Abstract

Breast cancer (BC) remains a leading cause of cancer-related deaths globally, with the tumor microenvironment (TME) playing a pivotal role in disease progression. Neutrophils, the most abundant white blood cells, have gained attention for their dualistic role in cancer immunity. Two major neutrophil subtypes, N1 and N2, have been identified, each exhibiting distinct functions in the TME. N1 neutrophils are typically associated with anti-tumor immunity, promoting tumor cell clearance through mechanisms such as reactive oxygen species production, cytokine release, and the activation of cytotoxic immune cells. In contrast, N2 neutrophils promote tumor progression, metastasis, and immune suppression by secreting pro-angiogenic factors and recruiting regulatory immune cells like Tregs and myeloid-derived suppressor cells. The polarization of neutrophils into N1 or N2 subtypes is regulated by the dynamic interactions within the TME, including cytokines, hypoxic conditions, and signals from tumor cells. In BC, factors such as IL-8, transforming growth factor-beta, and granulocyte-macrophage colony-stimulating factor drive N2 polarization, contributing to tumor evasion of immune surveillance. Conversely, pro-inflammatory signals can induce N1 polarization, which is often linked to favorable clinical outcomes. However, in aggressive breast cancer subtypes such as triple-negative breast cancer, the TME is more conducive to N2 polarization, resulting in poor prognosis and resistance to treatment.

## Introduction

Breast cancer (BC) remains one of the most prevalent and lethal cancers worldwide, with significant challenges in treatment and prognosis. Despite advances in early detection and therapies, such as chemotherapy, radiation, and targeted therapies, BC remains a major cause of morbidity and mortality, particularly in advanced stages or in aggressive subtypes like triple-negative breast cancer (TNBC). The complexity of the tumor microenvironment (TME) has emerged as a critical factor in understanding BC progression and therapeutic resistance. Within the TME, immune cells play a pivotal role in influencing tumor growth, metastasis, and response to treatment. Among these, neutrophils have recently garnered attention due to their dual roles in both promoting and inhibiting tumor development, depending on their polarization^[1–4]^. Neutrophils are the most abundant type of white blood cells and are typically associated with the innate immune response, primarily involved in defending against infections. However, research over the past decade has revealed that neutrophils also contribute significantly to cancer biology, with their functions being modulated by the signals they encounter in the TME. These signals can cause neutrophils to differentiate into two distinct subtypes: N1 and N2 neutrophils. N1 neutrophils are generally associated with anti-tumor immune responses, whereas N2 neutrophils have been implicated in promoting tumor progression, metastasis, and immune suppression. The balance between these two subtypes is believed to be crucial in determining the outcome of cancer progression^[5,6]^. The concept of neutrophil polarization into N1 and N2 subtypes has expanded our understanding of the role these cells play in cancer immunity. N1 neutrophils exhibit pro-inflammatory characteristics and are typically associated with a cytotoxic immune response. They produce reactive oxygen species (ROS), pro-inflammatory cytokines such as TNF-α, IL-1β, and IL-6, and other immune factors that activate cytotoxic T cells, natural killer (NK) cells, and other immune components. These actions enable N1 neutrophils to directly attack and kill tumor cells, as well as promote the recruitment of additional immune cells to the TME, enhancing overall anti-tumor immunity[7].

On the other hand, N2 neutrophils display immune suppressive properties and are often associated with tumor progression. These cells promote immune evasion by secreting cytokines and growth factors such as transforming growth factor-beta (TGF-β), vascular endothelial growth factor, and IL-10, which facilitate tumor cell migration, angiogenesis, and metastasis. Additionally, N2 neutrophils contribute to the recruitment and polarization of regulatory T cells (Tregs) and myeloid-derived suppressor cells (MDSCs), further dampening the immune response. Their secretion of pro-angiogenic factors accelerates the growth of new blood vessels, supporting the tumor’s ability to expand and invade surrounding tissues[8]. The role of neutrophils in BC has become increasingly significant as more is understood about the TME and its influence on immune cell function. The presence of N2 neutrophils in the TME has been linked to poor prognosis in BC patients. These cells not only support tumor growth but also contribute to resistance against conventional therapies like chemotherapy and immunotherapy. In aggressive subtypes such as TNBC, which lacks targeted therapy options and often presents with high metastatic potential, N2 neutrophils play a particularly detrimental role in supporting the cancer’s immune evasion and spread[9]. In contrast, N1 neutrophils have been shown to contribute to better clinical outcomes, particularly in cases where they are recruited to the tumor site in response to immune-modulatory treatments or therapies that aim to activate anti-tumor immunity. The presence of N1 neutrophils in the TME is associated with enhanced cytotoxic activity, suppression of tumor metastasis, and improved patient survival rates. The ability to modulate the polarization of neutrophils from the N2 to the N1 subtype represents a promising strategy for enhancing the effectiveness of BC treatments, especially in cases resistant to traditional therapies[10].

## Aim

The aim of this review is to explore the functional roles of N1 and N2 neutrophil subtypes in BC, examining their contributions to tumor progression, immune modulation, and therapeutic response.

## Rationale

The immune system plays a pivotal role in the progression and treatment of BC, with neutrophils emerging as key players within the TME. Neutrophils are traditionally viewed as components of the innate immune system with anti-tumor potential; however, emerging evidence reveals that their function is highly context-dependent and influenced by polarization into two distinct subtypes: N1 and N2. N1 neutrophils exhibit anti-tumor properties, while N2 neutrophils promote tumor growth, metastasis, and immune evasion. Given their dual roles, understanding and manipulating neutrophil polarization offers a promising avenue for therapeutic intervention in BC. BC remains one of the leading causes of cancer-related mortality worldwide, with significant challenges in overcoming therapy resistance, metastasis, and immune suppression. Despite advancements in treatment modalities such as chemotherapy, immunotherapy, and targeted therapies, the TME remains a critical factor in determining therapeutic outcomes. The TME often harbors an immunosuppressive milieu dominated by N2 neutrophils, which actively contribute to treatment resistance and tumor progression. By targeting the balance of neutrophil subtypes within the TME, it may be possible to shift the immune response towards a more anti-tumorigenic phenotype, enhancing the effectiveness of conventional treatments and improving patient survival rates. The rationale behind this review is to provide a comprehensive overview of neutrophil biology in BC, specifically focusing on the functional implications of N1 and N2 neutrophils and their polarization. By examining the molecular mechanisms that drive neutrophil behavior in the TME, this review seeks to identify potential therapeutic strategies that target neutrophil polarization to improve clinical outcomes. Furthermore, neutrophil subtypes may serve as valuable biomarkers for predicting treatment response and prognosis, thus enabling more personalized and effective BC therapies. This review aims to bridge the gap between fundamental immunology and clinical oncology by highlighting the therapeutic potential of neutrophil modulation in BC.

**HIGHLIGHTS**

- N1 neutrophils exhibit anti-tumor properties by promoting cytotoxicity, enhancing immune activation, and inhibiting tumor growth.
- N2 neutrophils support tumor progression through immunosuppression, angiogenesis, and facilitation of metastasis.
- Neutrophil polarization into N1 or N2 subtypes is influenced by cytokines like transforming growth factor-beta and IFN-β in the tumor microenvironment.
- The N1/N2 balance correlates with clinical outcomes, serving as a potential prognostic marker in breast cancer management.
- Therapeutic modulation of neutrophil phenotypes offers novel avenues for targeted breast cancer immunotherapy and improved patient outcomes.

## Review methodology

### Search strategy and selection criteria

A comprehensive literature search was conducted to identify relevant studies on the role of neutrophil subtypes (N1 and N2) in BC, focusing on their functional characteristics, polarization mechanisms, and clinical implications. The search included peer-reviewed articles, clinical trials, and reviews published in English from the last two decades (2003–2024) across several academic databases, including PubMed, Scopus, Web of Science, and Google Scholar. Keywords used in the search included “N1 and N2 neutrophils,” “neutrophil polarization in breast cancer,” “tumor microenvironment,” “neutrophils and immune response,” and “neutrophils and cancer therapy.”

Inclusion criteria for the review were:

- Studies investigating neutrophil function and polarization in BC
- Articles focusing on N1 and N2 neutrophils and their effects on tumor progression and immune modulation
- Studies assessing neutrophils as biomarkers or therapeutic targets in cancer
- Clinical studies, pre-clinical studies, and systematic reviews
- Studies published in peer-reviewed journals

Exclusion criteria included:

- Non-English language publications
- Studies not specific to BC or neutrophil biology
- Articles that did not provide functional or mechanistic insights into N1 and N2 neutrophil subtypes

### Limitations

This narrative review provides a synthesized overview of the emerging roles of N1 and N2 neutrophil subtypes in BC progression and treatment response. However, several limitations should be acknowledged:

1. Narrative review design: As a narrative review, this article may be subject to selection bias and lacks the methodological rigor of systematic reviews or meta-analyses. The inclusion of studies was not based on formal criteria or quantitative synthesis, which may affect the reproducibility and comprehensiveness of the findings.
2. Limited clinical trial evidence: Much of the current understanding of neutrophil polarization in BC is derived from preclinical studies, especially murine models. There is a significant lack of high-level clinical trial data directly evaluating the modulation of neutrophil subtypes in human BC patients, limiting the immediate translational applicability of proposed therapeutic strategies.
3. Translational gap from preclinical models: The extrapolation of findings from animal models to human disease remains a challenge due to interspecies differences in neutrophil biology, TME, and immune regulation. As a result, insights into N1/N2 dynamics in murine systems may not fully capture the complexity of human BC.
4. Incomplete characterization of human neutrophil subtypes: The phenotypic and functional characterization of N1 and N2 neutrophils in humans is still evolving. A lack of standardized markers and functional assays may lead to inconsistencies in subtype identification and classification across studies.

### N1 and N2 neutrophil subtypes

Neutrophils are a crucial component of the innate immune system, serving as the body’s first line of defense against pathogens. However, their roles in cancer biology, particularly in the TME, have garnered significant attention in recent years. Neutrophils can polarize into two distinct subtypes: N1 and N2, each exhibiting unique functional characteristics that either support or hinder tumor progression. Understanding the functional differences between these subtypes is critical for developing new therapeutic strategies in BC.

### N1 neutrophils: anti-tumor characteristics

N1 neutrophils are typically associated with anti-tumor immunity. These cells exhibit pro-inflammatory functions that contribute to the elimination of tumor cells and the activation of other immune responses. One of the key features of N1 neutrophils is their production of ROS, which induce direct cytotoxicity in tumor cells. ROS can damage DNA, proteins, and lipids within tumor cells, leading to their apoptosis or necrosis. In addition to ROS, N1 neutrophils release a variety of pro-inflammatory cytokines, such as TNF-α, IL-1β, IL-6, and IL-12, which can further enhance the immune response by promoting the activation of cytotoxic T lymphocytes, NK cells, and dendritic cells. These cytokines help recruit and activate other immune cells, which work in concert to attack the tumor. Furthermore, N1 neutrophils secrete chemokines such as CXCL8 (IL-8), which attract more immune cells to the tumor site, enhancing the anti-tumor immune response^[11–13]^. Another important function of N1 neutrophils is their ability to present antigens to T cells, thereby facilitating the activation of adaptive immunity. This characteristic makes N1 neutrophils particularly effective in coordinating the immune system’s response to tumor cells. In some cancer models, the presence of N1 neutrophils correlates with a better prognosis, as their activity promotes the clearance of tumor cells and impedes metastatic spread. In BC, studies have shown that high levels of N1 neutrophils are associated with a more favorable outcome, as these cells contribute to inhibiting tumor growth and enhancing the overall immune response^[14,15]^.

### N2 neutrophils: tumor-promoting characteristics

In contrast, N2 neutrophils are generally associated with tumor progression, immune suppression, and metastasis. These cells exhibit immune-suppressive functions that support the tumor’s ability to evade immune surveillance. N2 neutrophils are often characterized by their secretion of anti-inflammatory cytokines such as TGF-β, IL-10, and vascular endothelial growth factor (VEGF). These cytokines not only promote immune tolerance but also contribute to the tumor’s growth and spread. For example, VEGF is a potent angiogenic factor that stimulates the formation of new blood vessels, facilitating tumor expansion and metastasis. In addition, IL-10 and TGF-β can inhibit the function of cytotoxic T cells and NK cells, two key effectors in the immune system’s attack on tumor cells^[16–18]^. N2 neutrophils also play a role in immunosuppressive cell recruitment. They help recruit regulatory T cells (Tregs) and MDSCs, both of which dampen anti-tumor immunity. Tregs suppress the activity of cytotoxic T cells and NK cells, while MDSCs inhibit the immune system’s ability to respond effectively to tumor cells. Furthermore, N2 neutrophils secrete metalloproteinases (MMPs) that degrade the extracellular matrix (ECM), facilitating tumor cell invasion and metastasis. These factors combined enable N2 neutrophils to promote an immunosuppressive and pro-tumorigenic environment, which is often observed in more aggressive BC subtypes, such as TNBC^[15,19]^.

### Balance between N1 and N2 polarization

**Figure 1.**
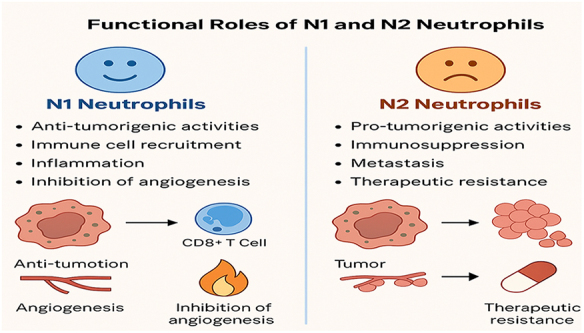
Functional roles of N1 and N2 neutrophils in tumor progression, immune modulation, and therapeutic response.

**Figure 2.**
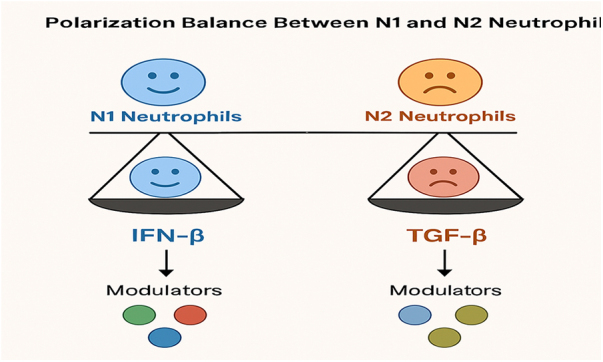
The polarization mechanisms and balance between N1 and N2 neutrophils, highlighting the influence of TGF-β and IFN-β, among other modulators.

The polarization of neutrophils into either the N1 or N2 subtype is not fixed; rather, it is influenced by various factors in the TME. Tumor-derived cytokines and growth factors, hypoxic conditions, and the presence of immune cells within the TME play a pivotal role in shaping neutrophil polarization. For instance, cytokines such as IL-8, granulocyte-macrophage colony-stimulating factor (GM-CSF), and G-CSF promote N2 polarization, whereas pro-inflammatory signals, including interferon-gamma (IFN-γ) and TNF-α, tend to favor N1 polarization. The balance between these subtypes is crucial, as it determines whether the immune response will be anti-tumorigenic or tumor-promoting^[20–22]^. In BC, the shift from an N1 to an N2-dominant neutrophil population is often associated with tumor progression, immune evasion, and resistance to therapy. For example, in TNBC, the TME tends to favor N2 polarization, contributing to the aggressive nature of the disease and its poor prognosis. In contrast, in less aggressive BC subtypes, the TME may promote the development of N1 neutrophils, which are associated with better outcomes. Modulating the balance between N1 and N2 neutrophils, through either targeted therapies or immune modulation, holds significant potential in improving the effectiveness of BC treatment (Figs. 1 and 2)^[9,23,24]^.

### The TME and neutrophil polarization in BC

The tumor microenvironment (TME) plays a crucial role in shaping the progression and therapeutic resistance of BC. Comprising various cell types, including tumor cells, immune cells, endothelial cells, fibroblasts, and ECM components, the TME provides the necessary support for tumor growth, invasion, and metastasis. One of the most significant players within the TME is immune cells, particularly neutrophils. As a key component of the innate immune system, neutrophils are involved in both tumor suppression and promotion, depending on their polarization into two distinct subtypes – N1 and N2. The polarization of neutrophils in the TME is influenced by a complex interplay of factors that either enhance anti-tumor immunity or facilitate immune evasion and tumor progression^[25,26]^. Neutrophil polarization into N1 or N2 subtypes is not a random process, but rather is driven by specific signals from the TME. These signals are often tumor-derived cytokines, growth factors, hypoxic conditions, and interactions with other immune cells. N1 neutrophils, which exhibit anti-tumor properties, are typically induced by pro-inflammatory signals, such as IFN-γ, TNF-α, and other cytokines associated with Th1 responses. These signals promote a pro-inflammatory state in neutrophils, leading to the release of ROS, pro-inflammatory cytokines (such as IL-1β, IL-6, and IL-12), and chemokines like CXCL8 (IL-8). These molecules contribute to tumor cell killing, activation of cytotoxic T cells and NK cells, and the recruitment of additional immune cells to the tumor site, all of which promote anti-tumor immunity^[19,27]^. Conversely, N2 neutrophils, which are associated with tumor progression and immune suppression, are typically driven by signals such as IL-8, GM-CSF, TGF-β, and VEGF. These cytokines and growth factors favor the polarization of neutrophils toward an immune-suppressive phenotype that promotes tumor growth, metastasis, and angiogenesis. N2 neutrophils secrete anti-inflammatory cytokines like IL-10 and TGF-β, which inhibit the activation and function of cytotoxic immune cells such as CD8+ T cells and NK cells. Moreover, N2 neutrophils recruit immune-suppressive cells, including regulatory T cells (Tregs) and MDSCs, to the TME, further dampening the anti-tumor immune response[28].

**Figure 3.**
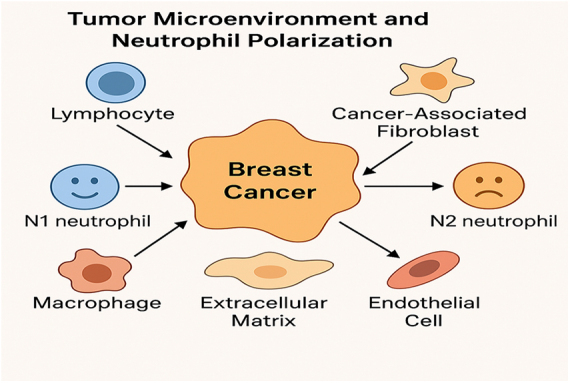
The breast cancer tumor microenvironment (TME) and its role in neutrophil polarization.

The dynamic nature of neutrophil polarization within the TME is influenced by a variety of factors, including hypoxia, nutrient availability, and the presence of tumor-associated macrophages (TAMs), cancer-associated fibroblasts (CAFs), and other stromal cells. Hypoxic conditions, which are common in solid tumors, promote the polarization of neutrophils toward the N2 phenotype through the activation of hypoxia-inducible factors. These transcription factors are known to regulate the expression of various genes involved in angiogenesis and immune suppression, such as VEGF and TGF-β. Additionally, CAFs and TAMs secrete cytokines and growth factors that further promote N2 polarization and sustain the immunosuppressive TME. The presence of these factors in BC contributes to an environment that favors tumor growth, resistance to therapy, and metastasis[29]. BC subtypes differ in the composition and characteristics of their TME, which influences neutrophil polarization and its subsequent effects on tumor progression. In more aggressive subtypes of BC, such as TNBC, the TME is often characterized by a higher proportion of N2 neutrophils. TNBC is known for its lack of targeted therapy options and its aggressive metastatic behavior, making the presence of N2 neutrophils particularly detrimental. These cells support tumor invasion, angiogenesis, and immune evasion, contributing to the poor prognosis associated with TNBC. In contrast, less aggressive subtypes, such as estrogen receptor-positive BC, may have a more balanced immune environment, with a greater presence of N1 neutrophils, leading to enhanced anti-tumor responses and better clinical outcomes[30]. The role of neutrophil polarization in BC highlights the importance of the TME in regulating immune responses. Inflammatory cytokines and growth factors produced by tumor cells and stromal components dictate the polarization of neutrophils, which in turn influences tumor progression and response to therapy. The shift from an anti-tumor N1 phenotype to a tumor-promoting N2 phenotype is a hallmark of an immune-suppressive TME, which facilitates tumor growth, metastasis, and resistance to chemotherapy and immunotherapy. Targeting the signals that drive neutrophil polarization or directly modulating neutrophil function presents a promising strategy for improving BC therapies. Additionally, the use of neutrophil subtypes as biomarkers for patient stratification and prognosis could help identify individuals most likely to benefit from specific treatment regimens (Fig. 3)[31].

### Clinical implications: targeting neutrophil subtypes in BC

**Figure 4.**
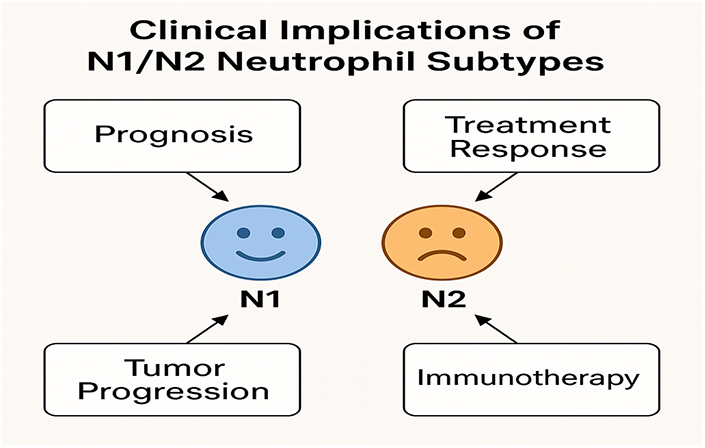
The clinical implications of N1/N2 neutrophil subtypes.

The polarization of neutrophils into the N1 (anti-tumor) and N2 (pro-tumor) subtypes within the TME significantly influences the progression and therapeutic response of BC. As the role of neutrophils in the TME becomes increasingly recognized, the potential to manipulate these cells for therapeutic benefit in BC is gaining attention. Targeting the balance between N1 and N2 neutrophils could offer a novel approach to enhancing anti-tumor immunity, reducing immune suppression, and improving the effectiveness of conventional and emerging therapies. Understanding the clinical implications of neutrophil subtypes offers new avenues for therapeutic intervention that could complement existing treatments such as chemotherapy, immunotherapy, and targeted therapies (Fig. 4)^[32,33]^.

### Modulating neutrophil polarization for immunotherapy

Immunotherapy has revolutionized the treatment of various cancers, including BC, by enhancing the body’s immune system to recognize and eliminate tumor cells. However, many patients, particularly those with aggressive subtypes like TNBC, fail to respond to immunotherapeutic agents. One reason for this lack of response is the immune-suppressive TME, which is often dominated by N2 neutrophils. These cells secrete cytokines such as TGF-β and IL-10, which dampen the activity of cytotoxic T cells and NK cells, key effectors in immunotherapy. By targeting the signaling pathways that promote N2 polarization, it may be possible to reprogram neutrophils toward a more anti-tumor N1 phenotype, thereby enhancing the effectiveness of immunotherapies^[34,35]^. For example, therapies that block key pro-tumor cytokines like IL-8, GM-CSF, or VEGF, which are associated with N2 polarization, could shift the neutrophil population in the TME toward an N1 phenotype. Additionally, agents that promote N1 polarization, such as IFN-γ or TNF-α, could be employed to stimulate neutrophils to become more pro-inflammatory and cytotoxic. A combination of immunotherapy with neutrophil-targeting agents could therefore provide a synergistic effect, enhancing the immune response against the tumor while overcoming immune evasion mechanisms driven by N2 neutrophils. Clinical trials exploring such combinatory strategies are needed to determine their safety and efficacy in BC treatment[36].

### Neutrophil subtypes as biomarkers for prognosis and treatment stratification

Neutrophil subtypes, particularly the balance between N1 and N2 neutrophils, may serve as valuable biomarkers for prognosis and treatment stratification in BC patients. The presence of a dominant N1 population in the TME has been associated with better prognosis and improved response to treatment, whereas a predominance of N2 neutrophils often correlates with worse outcomes, increased metastasis, and resistance to therapy. By analyzing the neutrophil phenotype in tumor biopsies or peripheral blood, clinicians may be able to assess the immunological status of a patient’s TME and predict their likelihood of responding to specific therapies. This approach could help tailor treatment regimens to individual patients, allowing for more personalized and effective care[37]. Moreover, neutrophil polarization could serve as an early indicator of treatment response. Changes in the proportion of N1 and N2 neutrophils following chemotherapy or immunotherapy could be monitored to assess the effectiveness of a given treatment. If therapy induces a shift from an N2 to an N1-dominant neutrophil population, it may signal an enhanced immune response and better tumor control. Conversely, if treatment results in an increase in N2 neutrophils, it may indicate immune evasion and resistance to therapy, prompting clinicians to adjust the treatment plan. The ability to track neutrophil subtypes in real-time could thus provide valuable insights into tumor dynamics and treatment efficacy[38].

### Overcoming neutrophil-mediated resistance to chemotherapy and targeted therapies

Chemotherapy and targeted therapies remain the cornerstone of BC treatment, yet resistance to these therapies is a major challenge. N2 neutrophils contribute to resistance by promoting an immunosuppressive environment that protects tumor cells from therapeutic attacks. They can enhance angiogenesis, tumor cell invasion, and the recruitment of immunosuppressive cells like Tregs and MDSCs, all of which contribute to therapeutic failure. Targeting N2 neutrophils or their associated cytokines could therefore help reverse therapy resistance. For instance, inhibitors of VEGF or other angiogenic factors produced by N2 neutrophils could reduce tumor vascularization and improve the efficacy of chemotherapy. Additionally, strategies aimed at blocking immune checkpoints, such as PD-1/PD-L1, which are often upregulated in the presence of N2 neutrophils, could restore immune surveillance and enhance the anti-tumor activity of both chemotherapy and targeted therapies^[39–41]^. One approach to overcoming neutrophil-mediated resistance could involve the use of small molecules or monoclonal antibodies that selectively target N2 neutrophils or the pro-tumor cytokines they produce. For example, neutralizing antibodies against IL-10, TGF-β, or CXCR2, which are critical for N2 polarization and tumor progression, may help reverse the immunosuppressive TME and make tumors more susceptible to treatment. Similarly, boosting the anti-tumor functions of N1 neutrophils through cytokine therapy (e.g. IFN-γ) or gene-editing technologies that enhance neutrophil polarization could be explored to complement chemotherapy or targeted therapies. By reprogramming neutrophils to shift toward an N1 phenotype, clinicians may improve the overall therapeutic response and reduce the likelihood of resistance in BC patients^[42–44]^.

### Neutrophils in BC metastasis: a new therapeutic target

Metastasis remains the leading cause of BC-related mortality, and neutrophils play a significant role in promoting tumor dissemination. N2 neutrophils contribute to metastasis by facilitating the invasion of tumor cells into surrounding tissues, promoting angiogenesis, and modifying the ECM. By secreting MMPs and other proteolytic enzymes, N2 neutrophils degrade the ECM and create a permissive environment for tumor cell migration. Targeting neutrophil polarization could be an effective strategy to prevent or slow down metastasis. By inhibiting the recruitment or activation of N2 neutrophils, it may be possible to limit tumor spread and improve patient survival[45]. Additionally, research into neutrophil-targeted therapies that disrupt the interactions between neutrophils and metastatic tumor cells could provide new avenues for preventing BC metastasis. For example, blocking the CXCL8-CXCR1/2 signaling axis, which is essential for neutrophil recruitment to metastatic sites, could impair the ability of N2 neutrophils to support tumor spread. Furthermore, therapies that modulate the polarization of neutrophils in the metastatic niche could shift the immune environment toward a more anti-tumorigenic phenotype, inhibiting the establishment and growth of metastatic colonies. As the understanding of neutrophils in metastasis deepens, new therapeutic strategies targeting neutrophil functions may emerge as an essential component of metastatic BC management^[46,47]^.

### Challenges and future directions

Despite significant advances in understanding the roles of N1 and N2 neutrophil subtypes in BC, several key challenges hinder their full clinical exploitation. These limitations also offer valuable direction for future research and therapeutic innovation.

#### Incomplete phenotypic characterization

Current methodologies lack definitive surface markers that can distinctly and reliably differentiate N1 from N2 neutrophils in human samples. Most knowledge derives from murine models, which limits translational applicability. Advancing single-cell transcriptomic and proteomic profiling is essential to clarify subtype-specific signatures and functions in human BC.

#### Plasticity and dynamic polarization

Neutrophils exhibit remarkable plasticity, transitioning between N1 and N2 phenotypes depending on environmental cues. This dynamic behavior complicates their therapeutic targeting and may contribute to treatment resistance. Developing interventions that stabilize the antitumor N1 phenotype without impairing host defense mechanisms remains a key challenge[48].

#### TME complexity

The TME’s heterogeneity and influence on neutrophil polarization through mediators such as TGF-β, IL-6, and hypoxia are not yet fully mapped. A deeper understanding of how the spatial and temporal dynamics of the TME shape neutrophil behavior will aid in designing targeted therapies.

#### Lack of clinical trials targeting neutrophils

To date, clinical trials directly targeting neutrophil subtypes or their polarization in BC are limited. More preclinical validation and translational studies are needed to assess the safety and efficacy of therapies that modulate neutrophil activity, such as TGF-β inhibitors or IFN-β agonists.

#### Immunotherapy integration

Neutrophil polarization significantly affects the tumor immune landscape, yet their integration into current immunotherapeutic strategies remains rudimentary. Combining neutrophil-modulating agents with checkpoint inhibitors or cancer vaccines may enhance efficacy, but optimal combinations and timing need to be defined[49].

### Future directions

- Biomarker development: Establishing reliable biomarkers to monitor neutrophil polarization in real time.
- Therapeutic reprogramming: Exploring reprogramming strategies to convert pro-tumoral N2 neutrophils into antitumoral N1 phenotypes.
- Personalized medicine: Tailoring neutrophil-targeted therapies based on individual tumor and immune profiles.
- Interdisciplinary research: Integrating systems biology, computational modeling, and spatial omics to unravel the neutrophil–TME interactions at a systems level.

## Conclusion

The dual roles of neutrophils in BC, as both tumor-promoting and tumor-fighting cells, underscore their importance in cancer progression and therapy response. The polarization of neutrophils into the N1 and N2 subtypes plays a crucial role in determining the immune landscape of the TME. While N1 neutrophils exhibit anti-tumor properties by enhancing immune surveillance and promoting cytotoxic activity, N2 neutrophils contribute to immune suppression, metastasis, and therapy resistance. Understanding the mechanisms underlying neutrophil polarization and the dynamic interactions within the TME offers novel therapeutic opportunities in BC treatment. Targeting the balance between N1 and N2 neutrophils provides a promising strategy for enhancing anti-tumor immunity and overcoming the limitations of current therapies, including chemotherapy, immunotherapy, and targeted therapies. By shifting the neutrophil phenotype toward an N1-dominant population, it may be possible to improve the effectiveness of existing treatments and reduce the incidence of metastasis and therapy resistance. Additionally, neutrophil subtypes can serve as valuable biomarkers for prognosis and treatment stratification, enabling personalized therapeutic approaches that optimize patient outcomes.

## Data Availability

Not applicable as this is a narrative review.

## Research registration unique identifying number (UIN)

Not applicable as this is a narrative review.
